# Risk Stratification of QTc Prolongations in Hospitalized Cardiology and Gastroenterology Patients Using the Tisdale Score—A Retrospective Analysis

**DOI:** 10.3390/jcm14020339

**Published:** 2025-01-08

**Authors:** Julian Steinbrech, Ute Amann, Michael Irlbeck, Sebastian Clauß, Dorothea Strobach

**Affiliations:** 1 Hospital Pharmacy, LMU University Hospital, 81377 Munich, Germany; 2 Doctoral Program Clinical Pharmacy, LMU University Hospital, 81377 Munich, Germany; 3 Faculty of Medicine, LMU, 81377 Munich, Germany; 4 Department of Anesthesiology, LMU University Hospital, 81377 Munich, Germany; 5 Department of Cardiology, LMU University Hospital, 81377 Munich, Germany; 6 DZHK (German Center for Cardiovascular Research), Partner Site Munich Heart Alliance, 81377 Munich, Germany; 7 Institute of Surgical Research at the Walter-Brendel-Center of Experimental Medicine, LMU University Hospital, 81377 Munich, Germany; 8 European Reference Network for Rare, Low Prevalence and Complex Diseases of the Heart (ERN GUARD-Heart), 81377 Munich, Germany; 9 Interfaculty Center for Endocrine and Cardiovascular Disease Network Modelling and Clinical Transfer (ICONLMU), LMU, 81377 Munich, Germany

**Keywords:** Long QT syndrome, risk assessment, pharmaceutical care, cardiology, gastroenterology

## Abstract

**Background/Objectives**: QTc prolongation can result in lethal arrhythmia. Risk scores like the Tisdale score can be used for risk stratification for targeted pharmaceutical interventions. However, the practical usability across different medical specialties has not been sufficiently investigated. The aim of this study was to compare relevant risk factors for QTc prolongation and to investigate the use of the Tisdale score in cardiology and gastroenterology patients. **Methods**: For patients on a cardiology and a gastroenterology ward receiving a weekly pharmaceutical electronic chart review, risk factors for QTc prolongation, QTc-prolonging drugs, and electrocardiograms (ECGs) were retrospectively collected for a four-month period (07-10/2023), and the Tisdale score and its sensitivity and specificity were calculated. **Results**: A total of 627 chart reviews (cases) (335 cardiology, 292 gastroenterology) were performed. The median age was 66 (range 20–94) years, and 39% (245) of patients were female. The presence of established risk factors (hypokalemia, renal impairment, age ≥ 68 years, cardiac diseases) differed significantly between the specialties. A median of 2 (range 0–5) QTc-prolonging drugs were prescribed in both groups. Baseline and follow-up ECG were recorded in 166 (50%) cardiology cases, of which prolonged QTc intervals were detected in 38 (23%) cases. In the 27 (9%) gastroenterology cases with baseline and follow-up ECG, no QTc prolongations were detected. Across both specialties, the Tisdale score achieved a sensitivity of 74% and a specificity of 30%. **Conclusions**: The presence of established risk factors for QTc prolongation differed significantly between cardiology and gastroenterology cases. The Tisdale score showed acceptable sensitivity for risk stratification; however, the limited availability of ECGs for gastroenterology cases was a limiting factor.

## 1. Introduction

QTc interval prolongation is a potentially severe adverse drug effect that can result in life-threatening torsade de pointes (TdP) arrhythmia [1,2,3,4]. There are currently more than 250 known drugs for which there is evidence of QTc prolongation [5]. Approximately 10% of the American population takes drugs with a risk of QTc prolongation [6]. In Germany, drugs with QTc-prolonging potential accounted for more than a fifth of the daily doses prescribed for outpatients in 2019 [7]. At hospital admission, studies from Germany and Italy found that as much as 50–89% of the patients were prescribed QTc-prolonging drugs [8,9]. A systematic review showed that 6.3% of patients exposed to QTc-prolonging drugs had a prolonged QTc interval, 2.6% developed ventricular arrhythmia, and 0.33% developed TdP [10]. QTc prolongation is particularly common in hospitalized patients. Approximately 24–28% of all patients in intensive care units are affected by QTc prolongation [1,3]. In patients of a geriatric ward, even 37% had a prolonged QTc interval [9].

Aside from drugs, various risk factors for QTc prolongation are known. These include, for example, cardiac diseases, electrolyte imbalances, female sex, and renal insufficiency [2,4,11]. Whereas the risk and the occurrence of QTc prolongation is well known for cardiology patients due to the presence of cardiac comorbidities, including heart failure, bradycardia, or myocardial infarction, the role in gastroenterology patients has not been studied as well [2,4,11]. There is, however, increasing evidence linking QTc prolongation to impaired liver function, such as liver cirrhosis or nonalcoholic fatty liver disease, as well as primary biliary cholangitis [12,13,14,15]. Overall, it is assumed that the risk of QTc prolongation is underestimated in clinical practice and that too little ECG (electrocardiogram)-monitoring is performed [2,11,16,17].

During the pharmaceutical care of hospitalized patients, warnings regarding QTc prolongations are frequently displayed in clinical decision support systems (CDSS) or drug interaction databases, especially when several QTc-prolonging drugs are combined. It is difficult for the pharmacist to determine whether or not a warning should be passed on to the physician in order to avoid over-alerting on the one hand but not to overlook important risks on the other. Possibilities for stratifying the patient-specific risk of QTc prolongation, such as risk scores, have recently been increasingly investigated in order to identify patients at risk at an early stage and thus prevent life-threatening arrhythmia [18]. Multiple risk scores were developed in different patient cohorts. The Tisdale risk score, which was originally developed in patients in cardiac intensive care units, has been investigated in various patient cohorts, leading to a reduction in the prescription of QTc-prolonging drugs, as well as a reduction in QTc prolongations overall [7,19,20,21,22,23,24,25,26]. However, there are several patient groups, like gastroenterology patients and patients on cardiology normal wards, where no risk score has yet been evaluated, even though these patients are often at risk for QTc prolongation [12,13,14,15], demonstrating the need for a viable stratification tool for pharmacists to manage the risk of QTc prolongation in clinical practice.

The aim of this study was to characterize the patient cohorts of a cardiology and a gastroenterology ward regarding the occurrence of established risk factors for QTc prolongation. Furthermore, we aimed to investigate the sensitivity and specificity of the Tisdale score as a screening-tool for pharmacists for risk stratification of QTc prolongations in two exemplary internal medicine wards during routine care.

## 2. Materials and Methods

In this monocentric retrospective study, we evaluated all adult patients in a cardiology and a gastroenterology general ward at the LMU hospital, a tertiary care university hospital in Munich, Germany, who received a routine weekly pharmaceutical electronic chart review in the period 07-10/2023. Electronic pharmaceutical chart reviews are regularly conducted using the electronic prescribing software Meona (Mesalvo GmbH, Freiburg, Germany, version number 2024.4.13), a computerized physician order entry–clinical decision support system (CPOE-CDSS). Data collection and evaluation were performed retrospectively. Ethics approval was obtained from the local Ethics Committee (24-0092).

Clinical and laboratory data were obtained from the electronic patient information system (SAP i.s.h.med, Cerner Corporation, North Kansas City, MI, USA). Patient medication was documented using the electronic prescribing software Meona. All data were collected for the day of the pharmaceutical chart review of a patient. This included risk factors for QTc prolongation according to the Tisdale risk score and laboratory data (most recent value, maximum 48 h prior to chart review). Furthermore, all QTc-prolonging drugs prescribed on the day of the pharmaceutical chart review and their classification according to CredibleMeds^®^ were documented [5]. ECGs during the inpatient stay, as well as the automatically calculated QTc interval, according to Bazett, were collected [27]. The Tisdale risk score (Table 1) was calculated retrospectively for the day of the pharmaceutical chart review.

Clinically relevant QTc prolongations in ECGs following the chart review (follow-up ECG) were defined as follows: QTc ≥ 500 ms or ΔQTc ≥ 60 ms in relation to the last ECG recorded before pharmaceutical chart review (maximum 7 days prior) (baseline ECG). In cases where the last ECG recorded before the chart review already showed a QTc ≥ 500 ms, QTc prolongation was defined as ΔQTc ≥ 60 ms in relation to this ECG [21].

Apart from the presence of a baseline or follow-up ECG, no data were missing for the investigated patients. For patients receiving more than one pharmaceutical chart review due to a longer stay or due to being admitted more than once during the study period, each chart review was evaluated as a separate event. This approach was chosen in order to reflect the usual care conditions as accurately as possible, where a risk of QTc prolongation of a patient would be evaluated at each chart review because risk factors such as hypokalemia or QTc-prolonging drugs are prone to change over the duration of a hospital stay.

Descriptive statistics were performed using Microsoft Excel 2016 (Seattle, WA, USA) and SPSS 2022 (IBM Corp., Armonk, NY, USA). Quantitative variables were reported as median and range and qualitative variables were reported using their frequency distribution. Significances for qualitative dichotomous variables were calculated using the Chi^2^-test. Significances for quantitative variables were calculated after testing for normal distribution using the Shapiro–Wilk test with the Student’s *t*-test (if data were distributed normally) or the Mann–Whitney-U test (if data were not distributed normally). A significance level of α = 0.05 was set. For cases where a baseline and a follow-up ECG were available, sensitivity and specificity, as well as positive and negative predictive values (PPV, NPV), were calculated for the Tisdale score regarding detected (Tisdale score ≥ 7) QTc prolongations in a follow-up ECG. Due to the study being a retrospective data analysis of a given patient cohort, which was predetermined by the time period chosen for data collection, sample size calculation was not possible.

## 3. Results

**Figure 1:**
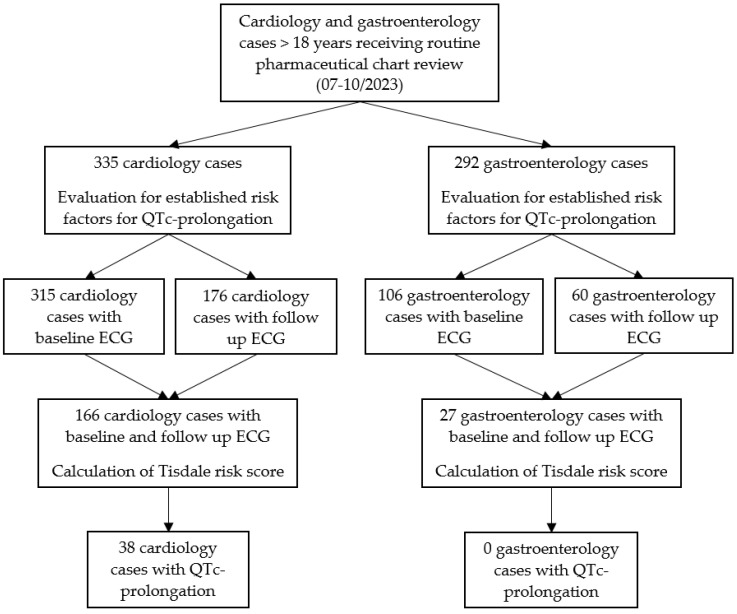
Study flow diagram.

In total, 627 pharmaceutical electronic chart reviews (cases) were performed in the study period across both specialties (335 cardiology, 292 gastroenterology) (Figure 1). The median age was 66 years (range 20–94 years), and 39.1% (245) of the cases were female. The 627 cases concerned 422 individual patients (230 cardiology, 192 gastroenterology). Repeated pharmaceutical evaluation was performed for 134 patients (71 cardiology, 63 gastroenterology) due to longer hospital stays or readmission.

Table 2 shows the occurrence of collected risk factors for QTc prolongation in cardiology and gastroenterology cases, the median Tisdale score, and the distribution of categories, as well as the calculated *p*-values.

Aside from the median number of QTc-prolonging drugs prescribed, the number of cases receiving at least two QTc-prolonging drugs, and the number of cases categorized as moderate risk according to the Tisdale score, all risk factors examined showed a statistically significant difference between cardiology and gastroenterology cases. Hypokalemia occurred significantly more often in gastroenterology cases. Of these 22 cases, 20 had a documented hepatic diagnosis (10 cases with liver cirrhosis, 10 cases with other diagnoses), and two cases were admitted due to acute diarrhea.

QTc-prolonging drugs were prescribed in 86.0% (539) of cases (median 2, range 0–5). A total of 82.1% (515) of cases received drugs classified as *Conditional Risk of TdP* according to CredibleMeds^®^, 16.9% (106) of drugs were classified as *Possible Risk of TdP*, and 12.0% (75) of drugs were classified in the highest risk category (*Known Risk of TdP*). Overall, 54 unique QTc-prolonging drugs were prescribed. The ten most commonly prescribed drugs and their frequencies are shown in Table 3. According to CredibleMeds^®^, there is evidence for QTc prolongation independent of the presence of other conditions for five of the ten most prescribed drugs across both specialties (*Known Risk of TdP* and *Possible Risk of TdP*).

A baseline ECG in the period of one week prior to chart review was available in 94.0% (315) of cardiology and 36.3% (106) of gastroenterology cases. A follow-up ECG in the period after the chart review was performed in 52.5% (176) of cardiology and 20.6% (60) of gastroenterology cases. However, only for 49.6% (166) of cardiology and 9.3% (27) gastroenterology cases a baseline ECG and a follow-up ECG were available. Of these, QTc prolongations were detected in 38 (22.9%) cardiology cases and none of the gastroenterology cases.

Table 4 shows the occurrence of investigated risk factors for QTc prolongation, the median Tisdale score, and the distribution of categories, as well as the calculated *p*-values in those cases, where an ECG was available in the period of one week prior to the chart review, as well as in the period after the chart review.

Cases developing a QTc prolongation in the period after the pharmaceutical chart review had significantly longer hospital stays and a higher number of ECGs, and they were more likely to have a high risk Tisdale score compared with cases without QTc prolongation. Higher age, heart failure, and a QTc interval of ≥450 ms in an ECG in the period of 1 week prior to the chart review, although not statistically significant, occurred more frequently in cases developing QTc prolongation. Altogether, 38 cases developing QTc prolongation were correctly screened using the Tisdale score (moderate or high risk), whereas 10 cases developing QTc prolongation were incorrectly classified as low risk (Table 5).

Of the 10 cases where a QTc prolongation was detected, which were classified as low risk by the Tisdale score, nine cases fell into the risk category “≥68 years”, and eight cases were 80 years or older. A transcatheter aortic valve implantation (TAVI) was performed in four of 10 cases within 48 h of the registration of a QTc prolongation. In one case, quetiapine—an atypical antipsychotic drug with conditional risk of TdP—was initiated prior to the QTc prolongation.

For the subgroup of 193 cases where a baseline and a follow-up ECG were available, the Tisdale risk score achieved a sensitivity of 73.7%, a specificity of 29.7%, a PPV of 20.4%, and an NPV of 82.1%. In addition, sensitivity, specificity, PPV, and NPV of the Tisdale score were calculated for the first assessment of each individual patient (n = 422). In 136 patients with baseline and follow-up ECG, 26 QTc prolongations were detected. The Tisdale score achieved a sensitivity of 73.1%, a specificity of 22.8%, a PPV of 19.4%, and an NPV of 81.6%.

## 4. Discussion

### 4.1. Summary of Findings

This study compared risk factors for QTc prolongation and investigated the use of the Tisdale risk score in cardiology and gastroenterology patients on general wards receiving a weekly pharmaceutical electronic chart review. In a retrospective evaluation of 627 chart reviews, we found significant differences in the presence of risk factors between the medical specialties. QTc prolongations were detected in 38 (19.7%) cases where both baseline and follow-up ECGs were available. QTc prolongations occurred significantly more often in cases with a Tisdale score ≥ 11 (high risk), a longer inpatient stay, and in cases receiving more ECGs. QTc-prolonging drugs are frequently prescribed across both investigated specialties. In a subgroup of 193 cases, the Tisdale score achieved a sensitivity of 73.7% and a specificity of 29.7% regarding detected QTc prolongations in ECGs recorded in the period after chart review.

### 4.2. Risk Factors for QTc Prolongation and Frequency of Prescription of QTc-Prolonging Drugs

All risk factors examined differed significantly between cardiology and gastroenterology cases, apart from the median number of QTc-prolonging drugs prescribed, the number of cases receiving at least two QTc-prolonging drugs, and the number of cases categorized as moderate risk according to the Tisdale score. The risk factors “age ≥ 68 years”, as well as diagnoses of heart failure and acute myocardial infarction, occurred more frequently in cardiology cases, while there were more female cases, cases with sepsis, and cases with hypokalemia in gastroenterology. Hypokalemia is common in patients with liver disease due to various pathomechanisms and was associated with a hepatic diagnosis in 20/22 cases in this cohort [28]. Other studies investigating the Tisdale score in different patient populations found an even higher proportion of patients with hypokalemia at the time of score calculation, ranging from 22% (patients prescribed QTc-prolonging antibiotics) to 25% (patients across different medical specialties) to 40% (patients in a cardiac intensive care unit) [19,21,22,29]. This is remarkable in that hypokalemia is a risk factor promotion QTc interval prolongation, which is generally correctable.

Although the types of risk factors varied significantly between specialties, the presence of risk factors indicates an overall risk for QTc prolongation in both investigated groups. A risk for QTc prolongation is generally well recognized in cardiology patients due to cardiac comorbidities being established risk factors but is not as well documented for gastroenterology cases, as there are currently only a few studies linking QTc prolongations to some diseases of the hepatobiliary system [2,4,12,13,14,15]. We think the characterization of hepatic diseases as risk factors for QTc prolongation still requires intensive research. In addition, awareness of QTc prolongation as a complication of hepatic diseases needs to be raised, and more ECG monitoring needs to be established in clinical practice.

In the cardiology and gastroenterology cases investigated, QTc-prolonging drugs were frequently prescribed. The top 10 QTc-prolonging drugs included drugs with a known risk of TdP, namely amiodarone as the third most frequently prescribed drug in cardiology cases (9.3%) and fluconazole as the sixth most frequently prescribed drug in gastroenterology gases (3.8%). These results differ from findings for the outpatient setting in Germany, where the most frequently prescribed QTc-prolonging drugs were citalopram and escitalopram, while amiodarone was in third place [7]. Across both specialties, pantoprazole (55.2%) was the most frequently prescribed QTc-prolonging drug classified as *Conditional Risk of TdP*. Our findings are consistent with other studies in which proton pump inhibitors were also found to be one of the most often prescribed QTc-prolonging drugs in hospitals [8,9,30]. The use of proton pump inhibitors is suspected to be unjustified in many cases, and long-term use has been criticized for various adverse outcomes, even increased mortality [31,32].

### 4.3. Evaluation of the Tisdale Risk Score for Risk Stratification of QTc Prolongation

No single risk factor listed in the Tisdale score occurred significantly more frequently in cases with QTc prolongation in this patient cohort, which supports the hypothesis that QTc prolongations are multifactorial and cannot be measured or predicted by the use of only one parameter in clinical practice [33]. Therefore, the use of risk scores, including multiple parameters, such as the Tisdale score, seems to be a valid approach for the risk stratification of QTc prolongations. One benefit of the Tisdale score, in particular, is that it is easy to calculate, and the parameters needed for the calculation of the score are usually readily available in clinical practice. Other published QT risk scores (e.g., RISQ-Path score) are often complex and cannot be calculated by hand, or the availability of their many parameters is often poor [20,34]. In addition, most of them have not been studied as thoroughly in different patient groups or have not performed well outside the patient cohorts of their original development [20].

The Tisdale score showed an acceptable sensitivity for risk stratification in cardiology and gastroenterology cases with 73.7%, which is similar to the achieved sensitivity in the original patient cohort of intensive care cardiology patients (74% [19]). QTc prolongations are associated with a risk of life-threatening arrhythmia. Therefore, a high sensitivity of the risk score is important for the detection of patients at risk. Nevertheless, 10 cases in which QTc prolongation was registered were classified as low risk according to the Tisdale score. Of these, patients in nine cases were >68 years old, a risk factor that contributes to the Tisdale score. Interestingly, four patients underwent TAVI within 48 h prior to the registration of QTc prolongation. This is in contrast to a study by Kasapkara et al., which showed that TAVI is associated with a shortening of the QTc interval, as well as the QTc dispersion, even short-term [35], while Leire et al. found a prolongation of the QTc interval after TAVI [36]. TAVI as a potential risk factor for QTc prolongation should, therefore, be investigated in further studies.

The specificity of the Tisdale score in this study was 30%, which is considerably lower than the specificity achieved by Tisdale et al. (77% [19]). This could be due to the fact that the patient cohort investigated in this study differs substantially from the patient cohort studied by Tisdale et al. Potential reasons for the difference in specificity could be differences in the patient setting (intensive care versus normal ward) and the presence of risk factors for QTc prolongation. In the cohort of intensive care cardiology patients investigated by Tisdale et al., the risk factors female sex, acute myocardial infarction, sepsis, and the prescription of loop diuretics occurred more frequently, whereas QTc-prolonging drugs occurred less frequently than in the cohorts investigated in this study. The proportion of patients with heart failure was highest in the cardiology cases investigated in this study, followed by the Tisdale cohort, while heart failure occurred least commonly in the gastroenterology cases. Furthermore, all patients investigated by Tisdale et al. underwent daily ECG monitoring, resulting in a substantially higher proportion of patients with a baseline and follow-up ECG [19]. Low specificities (<50%) are also achieved by other QTc-risk scores, even in the respective patient cohorts where the scores were developed [20,34,37,38,39,40]. Many authors argue that a high specificity of the risk score can be neglected in favor of high sensitivity because interventions to manage the risk of QTc prolongation resulting from a false positive classification are usually non-invasive and inexpensive, such as ECG monitoring [34,37,38,41].

### 4.4. Limitations

Limitations of this study include the availability of ECGs, especially in gastroenterology cases. Many gastroenterology cases had to be excluded from the final evaluation because of a lack of ECGs during the inpatient stay. No additional ECGs could be obtained because of the retrospective nature of the study. However, established risk factors such as cardiac comorbidities, higher age, renal insufficiency, and QTc ≥ 450 ms in an ECG prior to chart review, as well as a high risk Tisdale score, occurred significantly less often in all gastroenterology cases in our study. It could, therefore, be argued that, despite the lack of ECGs, gastroenterology cases in this cohort had a lower overall risk for QTc prolongation than cardiology cases. Overall, it is well known that ECG monitoring is often not performed sufficiently in clinical practice. A meta-analysis of 14 studies from different disciplines showed that in the inpatient setting, ECGs were recorded in about 75% of patients before the initiation of drugs with a high risk of QTc prolongation, and recommended follow-up ECGs were performed in only about 39% [17]. This is an aspect that could be drastically improved by targeted pharmaceutical interventions in patients at risk, stratified by risk scores like the Tisdale score. One study already exists that showed that risk score-based stratification for QTc prolongations led to targeted ECG monitoring in patients at risk [22]. An improvement in ECG monitoring in patients at risk for QTc prolongations through pharmaceutical interventions could ultimately lead to an improvement in drug therapy safety.

The number of ECGs per case and the length of stay being significantly higher in cases where a QTc prolongation was detected may be related to the fact that intensified ECG monitoring was deemed to be important by the physicians because of a patient’s history of arrhythmia and led to a longer hospitalized stay. For example, patients receiving amiodarone in the LMU University Hospital cardiology ward undergo intensified ECG monitoring during the loading phase because of the known risk of QTc prolongations [5]. Furthermore, the patients evaluated in this study were receiving a pharmaceutical chart review as part of usual care, which might have led to an increased awareness of the patients at risk for QTc prolongation. Because ECGs were, therefore, possibly recorded more likely in patients at higher risk, an ECG-based selection bias cannot be ruled out.

In addition, considering each pharmaceutical chart review as an independent event may have led to an over-representation of data from certain patients, such as higher-risk patients who require a longer hospital stay. In clinical practice, pharmaceutical chart reviews are often performed more than once during a patient’s hospital stay, with the risk of QTc prolongation having to be assessed each time by the pharmacist. This is particularly important with regard to QTc prolongation, as many risk factors that contribute to QTc prolongation can change rapidly or often during a hospitalization (e.g., potassium levels, number of QTc-prolonging drugs, loop diuretic prescription). To reflect clinical practice and real-world conditions as closely as possible, this study chose to consider each pharmaceutical chart review as an individual event. Regarding the predictive parameters (sensitivity, specificity, PPV, NPV) of the Tisdale score, similar results were obtained when comparing the analysis of the chart review cases with the analysis of the individual patients, supporting the validity of the case-based approach.

Another possible limitation is the automatic measurement of the QTc interval. Some authors prefer manual measurement of the QTc interval because of possible misinterpretations of the QTc interval by the computer [42]. However, other studies showed that manually measured QTc intervals, especially when performed by non-cardiology physicians, are often not interpreted correctly [43,44]. As automatically measured QTc intervals are widely used in clinical practice, automatically measured QTc intervals were used for the purpose of this study [45].

### 4.5. Further Studies

Overall, risk scores are promising tools regarding the detection of patients at risk for QTc prolongations while reducing the over-alerting of the classical clinical decision support systems. Inclusion of the Tisdale score in clinical decision support systems, where the score is automatically calculated when QTc-prolonging drugs are prescribed, for example, resulted in a reduced risk of QTc prolongation, as well as a reduction in the prescription of QTc-prolonging drugs in a prospective study in cardiology patients [21]. This approach also led to a reduction in over-alerting associated with classical CDSS while generating more useful warnings regarding QTc prolongation for pharmacists and physicians [46]. Therefore, the performance of the Tisdale score and its use as a risk stratification tool for targeted pharmaceutical interventions should be further investigated in prospective studies in these and other patient cohorts.

Further studies evaluating the use of the Tisdale score in other patient cohorts could include multivariate logistic regression analysis to investigate the influence of individual risk factors for QTc prolongation in a specific patient group and to potentially modify the Tisdale score for these patient groups to achieve better predictive results. Due to the limited sample size and ECG availability, this approach could not be fulfilled in this study but should be considered in further studies evaluating the Tisdale score in different patient cohorts.

## 5. Conclusions

Risk stratification of QTc prolongations using the Tisdale risk score, which is distinguished by the simple calculation and the usually ready availability of the parameters required for the calculation showed an acceptable sensitivity in a cohort of cardiology and gastroenterology cases investigated in this study. Through the early identification of patients at risk, risk stratification using the Tisdale score aiding pharmacists in the generation of targeted interventions could lead to an improvement of ECG monitoring, ultimately resulting in an increase in drug therapy safety in these patient groups.

## Figures and Tables

**Table 1:** Tisdale risk score [19].

Parameter	Weight
Age ≥ 68 years	1
Female	1
Acute myocardial infarction	2
Heart failure	3
Sepsis	3
Potassium ≤ 3.5 mmol/L	2
Admission QTc ≥ 450 ms	2
Loop diuretics	1
1 QTc-prolonging drug	3
≥2 QTc-prolonging drugs	3
<7: Low risk	
7–10: Moderate risk	
≥11: High risk	

**Table 2:** Occurrence of risk factors for QTc prolongation in cardiology and gastroenterology cases and calculated Tisdale risk score.

	Cardiology	Gastroenterology	*p*
Number of chart reviews performed (cases)	335	292	
Female (n (%))	124 (37.0)	121 (41.4)	<0.001
Age [years] (median (range))	75 (25–94)	57 (20–92)	0.002
Duration of stay [days] (median (range))	9 (1–50)	11 (1–66)	<0.001
Age ≥ 68 years (n (%))	221 (66.0)	71 (24.3)	<0.001
eGFR < 60 mL/min/1.73 m^2^ (n (%))	172 (51.3)	90 (30.8)	<0.001
Heart failure (n (%))	117 (34.9)	4 (1.4)	<0.001
Acute myocardial infarction (n (%))	26 (7.8)	0 (0)	<0.001
Sepsis (n (%))	0 (0)	4 (1.4)	0.032
Potassium ≤ 3.5 mmol/L (n (%))	10 (3.0)	22 (7.5)	0.010
**ECG data**			
ECG available in the period of one week prior to chart review (n (%))	315 (94.0)	106 (36.3)	<0.001
Of which QTc ≥ 450 ms	184 (54.9)	51 (17.5)	<0.001
ECG available in the period after chart review (n (%))	176 (52.5)	60 (20.6)	<0.001
ECG available in the period of one week prior to and in the period after chart review (n (%))	166 (49.6)	27 (9.3)	<0.001
Of which with QTc-prolongation (n (%))	38 (22.9)	0 (0)	<0.001
Number of ECGs per case (median (range))	4 (0–29)	0 (0–8)	<0.001
**Drug data and Tisdale score**			
Number of QTc-prolonging drugs prescribed (median (range))	2 (0–5)	2 (0–5)	0.375
Prescription of ≥2 QTc-prolonging drugs (n (%))	180 (53.7)	155 (53.1)	0.871
Loop diuretic (n (%))	198 (59.1)	112 (38.4)	<0.001
Tisdale score (median (range))	9 (0–18)	6 (0–12)	<0.001
low risk (<7) (n (%))	105 (31.3)	151 (51.7)	<0.001
moderate risk (7–10) (n (%))	131 (39.1)	128 (43.8)	0.230
high risk (≥11) (n (%))	99 (29.6)	13 (4.5)	<0.001

ECG: electrocardiogram, eGFR: estimated glomerular filtration rate.

**Table 3:** Prescribed drugs with a risk of QTc prolongation according to CredibleMeds^®^ across all cases and stratified for cardiology and gastroenterology cases [5]. Drugs with a *Known Risk of TdP* are presented in bold, and drugs with a *Possible Risk of TdP* are in italics.

	Drug	Risk Classification	n (%)
**Overall**			
1	Pantoprazole	Conditional Risk of TdP	346 (55.2)
2	Torasemide	Conditional Risk of TdP	295 (47.1)
3	Piperacillin/Tazobactam	Conditional Risk of TdP	85 (13.6)
4	*Tacrolimus*	*Possible Risk of TdP*	*43 (6.9)*
5	Hydrochlorothiazide	Conditional Risk of TdP	34 (5.4)
6	**Amiodarone**	**Known Risk of TdP**	**32 (5.1)**
7	*Mirtazapine*	*Possible Risk of TdP*	*23 (3.7)*
8	Quetiapine	Conditional Risk of TdP	21 (3.4)
9	*Levetiracetam*	*Possible Risk of TdP*	*18 (2.9)*
10	**Fluconazole**	**Known Risk of TdP**	**14 (2.2)**
**Cardiology**			
1	Torasemide	Conditional Risk of TdP	190 (56.7)
2	Pantoprazole	Conditional Risk of TdP	163 (48.7)
3	**Amiodarone**	**Known Risk of TdP**	**31 (9.3)**
4	Hydrochlorothiazide	Conditional Risk of TdP	28 (8.4)
5	Piperacillin/Tazobactam	Conditional Risk of TdP	15 (4.5)
6	*Mirtazapine*	*Possible Risk of TdP*	*14 (4.2)*
7	Tacrolimus	Possible Risk of TdP	9 (2.7)
8	Quetiapine	Conditional Risk of TdP	9 (2.7)
9	*Levetiracetam*	*Possible Risk of TdP*	*8 (2.4)*
10	*Melperone*	*Possible Risk of TdP*	*7 (2.1)*
**Gastroenterology**			
1	Pantoprazole	Conditional Risk of TdP	183 (62.8)
2	Torasemide	Conditional Risk of TdP	105 (36.0)
3	Piperacillin/Tazobactam	Conditional Risk of TdP	70 (24.0)
4	*Tacrolimus*	*Possible Risk of TdP*	*34 (11.6)*
5	Quetiapine	Conditional Risk of TdP	12 (4.1)
6	**Fluconazole**	**Known Risk of TdP**	**11 (3.8)**
7	*Levetiracetam*	*Possible Risk of TdP*	*10 (3.4)*
8	*Mirtazapine*	*Possible Risk of TdP*	*9 (3.1)*
9	*Venlafaxine*	*Possible Risk of TdP*	*9 (3.1)*
10	Sertraline	Conditional Risk of TdP	9 (3.1)

**Table 4:** Cases with an ECG in the period of one week prior to (baseline) and in the period after chart review (follow-up) with and without QTc prolongation (n = 193).

	QTc Prolongation	No QTc Prolongation	*p*
Number of chart reviews performed (cases)	38	155	
Female (n (%))	16 (42.1)	70 (45.2)	0.734
Age [years] (median (range))	79.5 (34–94)	75 (27–92)	0.050
Duration of stay [days] (median (range))	13 (4–35)	9 (1–61)	0.034
Age ≥ 68 years (n (%))	27 (71.1)	101 (65.2)	0.491
eGFR < 60 mL/min/1.73 m^2^ (n (%))	23 (60.5)	81 (52.3)	0.360
Heart failure (n (%))	14 (36.8)	35 (22.6)	0.070
Acute myocardial infarction (n (%))	0 (0)	11 (7.1)	0.091
Sepsis (n (%))	0 (0)	0 (0)	n.a.
Potassium ≤ 3.5 mmol/L (n (%))	2 (5.3)	11 (7.1)	0.686
QTc ≥ 450 ms in an ECG in the period of 1 week prior to chart review	28 (73.7)	90 (58.1)	0.077
**ECG data**			
Number of ECGs per case (median (range))	9 (3–18)	5 (2–29)	<0.001
Number of QTc-prolonging drugs prescribed (median (range))	2 (0–3)	2 (0–5)	0.658
**Drug data and Tisdale score**			
Prescription of ≥2 QTc-prolonging drugs (n (%))	23 (60.5)	87 (56.1)	0.623
Loop diuretic (n (%))	26 (68.4)	83 (53.5)	0.097
Tisdale score (median (range))	10 (1–15)	9 (0–18)	0.194
low risk (<7) (n (%))	10 (26.3)	46 (29.7)	0.682
moderate risk (7–10) (n (%))	12 (31.6)	72 (46.5)	0.097
high risk (≥11) (n (%))	16 (42.1)	37 (23.9)	0.024

ECG: electrocardiogram, eGFR: estimated glomerular filtration rate.

**Table 5:** Cases with QTc prolongation and low risk Tisdale score.

Case Number	Identifiable Potential Risk Factors for QTc Prolongation and Cardiac Diseases
1	Age > 68 years, diabetes mellitus, obesity, coronary heart disease, aortic valve stenosis, TAVI (within 48 h before QTc prolongation)
2	Age > 68 years, diabetes mellitus, obesity, coronary heart disease, TAVI
3	Age > 68 years, eGFR < 30 mL/min/1.72 m^2^, coronary heart disease, atrial fibrillation, aortic valve stenosis, TAVI (within 48 h before QTc prolongation)
4	Age > 68 years, syncope, acute renal failure, quetiapine therapy initiation
5	Age > 68 years, supraventricular tachycardia
6	Age > 68 years, coronary heart disease, aortic valve stenosis, TAVI (within 48 h before QTc prolongation)
7	Age > 68 years, cardiac amyloidosis, atrial flutter, aortic valve stenosis
8	Heart failure, tricuspid valve stenosis, pacemaker (atrioventricular block)
9	Age > 68 years, aortic valve stenosis, TAVI (within 48 h before QTc prolongation)
10	Age > 68 years, coronary heart disease, aortic valve stenosis, tricuspid valve stenosis

TAVI: transcatheter aortic valve implantation, eGFR: estimated glomerular filtration rate.

## Data Availability

Data are available from the corresponding author upon reasonable request.
